# Effects of Farm Management Practices and Transport Time on Post-Mortem Changes of *Longissimus lumborum* Muscle Proteins in Suckling Goat Kids

**DOI:** 10.3390/foods9070934

**Published:** 2020-07-15

**Authors:** Tomás Francisco Martínez, María Jesús Alcalde, María Isabel Sáez, María Dolores Suárez

**Affiliations:** 1 Departamento de Biología y Geología, Universidad de Almería, 04120 Almería, Spain; tomas@ual.es (T.F.M.); msc880@ual.es (M.I.S.); 2 Departamento de Ciencias Agroforestales, Universidad de Sevilla, 41013 Sevilla, Spain; aldea@us.es

**Keywords:** farm management, goat kids, myofibrillar protein, sarcoplasmic protein, SDS-PAGE, transport

## Abstract

The combined effect of farm management practices, transport time, and ageing time on the electrophoretic changes of sarcoplasmic (SPP) and myofibrillar (MFP) protein fractions of goat kids was studied. A total of 64 suckling goat kids were withdrawn from two farms with “high” (GW) and “low” (DW) welfare-friendly management practices, and they were transported for 2 or 6 h immediately before slaughtering. *Longissimus lumborum* samples were obtained at 3, 8, and 21 days post-mortem, and muscle proteins were separated by sodium dodecyl sulfate polyacrylamide gel electrophoresis SDS-PAGE. Both protein extracts displayed significant changes attributable to meat maturation. Managing conditions of kids in DW farms increased the post-mortem susceptibility of muscle proteins. Some MFP of *Longissimus lumborum* muscle, such as troponin T, as well as 26–30 and 35–37 kDa fractions were influenced significantly by deficient on-farm management, and therefore, these protein fragments might be considered as indicators of low-welfare on-farm management in goat kids.

## 1. Introduction

Studies evaluating the potential impact of stressful management practices on the quality of animal products are of great interest [1]. Pre-slaughtering, handling, and transport of animals are acknowledged as major sources of stress, and thus, even under careful management, it is likely that conditions during transport might lead to catecholamine release and muscle glycogen depletion [2].

Other factors influencing meat quality are linked to post-mortem autolytic reactions, which are responsible for the disruption of muscle structures through the ageing process. Proteolysis of some myofibrillar components, such as Z line (α-actinin), as well as the degradation of regulatory proteins like troponin T and tropomyosin [3] are considered crucial for meat textural changes. Thereby, myofibrillar proteins (MFP) have been widely acknowledged as valuable indicators for estimating the extent of such changes throughout muscle ageing [4].

On the contrary, changes in sarcoplasmic proteins (SPP) have not received much attention when it comes to assessing muscle post-mortem changes. However, in the last few decades, the role of SPP as indicators of meat quality is resurging, given that most metabolic enzymes catalyzing post-mortem biochemical processes are sarcoplasmic proteins.

Numerous studies have also applied proteomic tools as two-dimensional polyacrylamide gel electrophoresis (2-D PAGE) and one dimensional electrophoresis (SDS-PAGE) to the analysis of proteins or metabolites in an attempt to describe changes in muscle proteins post-mortem [5,6,7,8,9]. In fact, a correlation between certain muscle protein fractions and meat tenderness has been described [10,11,12]. Nevertheless, electrophoretic separation of muscle protein fractions by the SDS-PAGE method still remains a simple, fast, and accurate procedure that enables and identification of protein fragments of interest as biomarkers in this regard. 

In the last few years, identification of biological markers indicative of stress status in farm animals has been highly studied. Thus, it has been described that metabolic changes in muscle cells caused by stress condition can affect certain structural (myosin light chain isoforms, two fast skeletal myosin light chain 2 isoforms, troponin T, actin) and enzymatic (protein kinase, glyceraldehyde-3-phosphate dehydrogenase, lactate dehydrogenase) muscle proteins, which can be revealed by proteomic analysis [6,13,14,15].

Although the physiological characteristics of goats enable them to succeed even in arid lands characterized by their scarcity of natural resources, in which no other livestock is viable, goat meat is in general less appreciated than sheep meat due to higher hardness, and lower growth rate and carcass yield. However, suckling kids slaughtered around 8–10 kg live weight are greatly appreciated in some Mediterranean countries such as Spain, owing to the fact that this meat is tender and flavorful, with low intramuscular and subcutaneous fat content [16,17]. 

In general, goats are very susceptible to stress, as indicated their low concentration of glycolytic intermediates both in muscle and blood, as well as by the high ultimate muscle pH measured in carcasses [18,19]. As mentioned above, transport is a very stressful management practice for domestic animals, and in this regard, valuable work is available in the literature focused on the effects of the transport on goats [20,21]. However, studies broaching the possible influence of long-term farm management on meat quality of goat kids are limited, despite the essential role that these animals play in Mediterranean smallholdings. Some literature is available on suckling lambs [2,22] but few studies have assessed together the influence of farm management practices and transport in suckling kids.

In a previous study [23] we have evaluated the influence of farming practices and transport time (2 or 6 h) on some physiological parameters linked to stress response, as well as on meat quality of suckling goat kids measured around slaughtering. The aim of the present work was the assessment of those factors on myofibrillar and sarcoplasmic protein fractions of *Longissimus lumborum* muscle throughout meat ageing, in order to identify their possible influence on sarcoplasmic and myofibrillar protein profile. 

## 2. Materials and Methods

### 2.1. Animal Handling and Slaughtering

The Universidad de Almería “Comité Ético de Experimentación Animal” approved all procedures concerning the animals managed in this study in compliance with European Union Directive (Directive 2010/63/EU), and with the Spanish Real Decreto (Real Decreto 53/2013). Animals were transported in accordance to European Council Regulation 1/2005. 

A sample of 64 male single-born suckling goat kids were utilized (10.05 ± 1.21 live BW, 30 to 36 days old) from Spanish Blanca Celtibérica breed (meat aptitude; FEAGAS, 2015) in Andalusia (37°48′ N 2°32′ W and ~953 m above sea level). The complete experimental design was described previously [23]. Briefly; 24 farms were previously evaluated to assess the quality of the welfare management practices using a survey of 36 items based on protocol for cattle Welfare Quality^®^ assessment [24]; indicators of goat on-farm welfare established by [25] were adapted to extensive systems.

The items were grouped into different categories related to pen environmental conditions, stocking density, health status (direct observation and disease and mortality records), and health management (e.g., prophylactic treatments), animal behavior, quality of grazing areas, feed supplementation for dams, breeding management, control of colostrum feeding, etc. A range between 0 and 50 points (the higher the score, the worse the managing practices) was established by the farms, and were classified into 3 categories, good (GW, good welfare level farm, from 0 to 5 points), average (ranging from 5 to 10), and deficient welfare level (DW, over 10 points). From these farms, two were selected for the experiment, going to good (GW; 2 points) and deficient (DW; 12 points) categories, respectively.

### 2.2. Experimental Design

Detailed experimental design was described in [23]. After reaching the target live weight (around 10 kg), a total of 64 animals from both farms were randomly selected, and grouped into four lots of 16 kids. Two of those lots (32 animals, corresponding to 2 farms × 2 transport times × 8 animal replicates) were transported on two consecutive days; that is, two complete runs were carried out.

Animals were loaded in a livestock lorry (0.2 m^2^ per each) in compliance with European Council Regulation 1/2005 on animal welfare during transport. Transport time was of 2 (ST) and 6 (LT) hours, usual distances average and maximum to the slaughterhouse in the area studied. Goat kids (thirty two) were transported during 2 h, and 16 (short transport group, ST) were discharged at the slaughterhouse, and the other 16 animals continued the journey for 4 supplementary hours (6 h, long transport, LT) until they reached the slaughterhouse. The complete process was repeated the next day with the remaining 32 animals. 

Slaughtering (carried out without lairage period) was performed by standard commercial procedures using manual electrical stunning), in compliance with Council Regulation EC 1099/2009 (250 V; 1.0 A for 1–3 s, stunning tongs). 

Subsequently, carcasses were suspended by the Achilles tendon and chilled during 24 h at 4 °C. From the right half of the carcass, the *Longissimus lumborum* muscle was dissected and the medium segment was stored under vacuum in a cold room at 4 °C. At 3, 8, and 21 days after slaughtering, samples were taken (4 per treatment) of approximately 1 cm^3^ each, frozen in liquid nitrogen, stored at −80 °C, and freeze-dried. 

### 2.3. Extraction of Sarcoplasmic (SPP) and Myofibrillar (MFP) Fractions

For SPP, stored and freeze-dried *Longissimus lumborum* samples were homogenized with a mechanical homogenizer (Polytron PT-2100, Kinematica AG, Lucerne, Switzerland) at 11,200 g for 1 min (1.5 mg muscle mL^−1^) in cool low ionic strength buffer (10 mM NaHCO3, 1mM EGTA, pH 7.2). The homogenates were centrifuged (10,000 *g*; 20 min; 4 °C) and the selected supernatants were extracted twice and pooled, constituting the SPP fraction. 

Remaining pellets were again extracted twice in chilled myofibrillar extracting buffer (8 M urea, 50 mM Tris-HCl, 2% *v*/*v* Triton X-100, 2 mM dithiotreitol, pH 7.2), and the pooled supernatants obtained were selected as MFP fraction. Tubes were kept in crushed ice throughout the entire homogenization process. 

### 2.4. Determination of Soluble Protein 

The soluble protein content in SPP extracts was determined by the Bradford method [26], at 595 nm. Due to the disturbances of detergents with this method, soluble protein was determined in MFP extracts (containing Triton X-100) by Biuret reaction [27] at 540 nm. In both methods was utilized bovine serum albumin as standard. 

### 2.5. Total Amino Acid Determination

Total free amino acid content in extracts were determined by the o-phthaldialdehyde (OPA) procedure [28]. Fifty µL of SPP extracts were mixed with the same amounts of 200 g L^−1^ TCA with the aim of precipitating soluble protein. The mix was centrifuged (20,000 *g*, 4 °C, 15 min) and 10 µL of supernatants were mixed with 1.0 mL of OPA reagent (50 mM sodium tetraborate, 1% *w*/*v* SDS, 8 mg L-1, 0.2% *v*/*v* β-mercaptoethanol) and incubated for 2 min at room temperature, then, the absorbance was determined in triplicate at 340 nm in a microplate reader (PowerWave XS, BIO-TEK, Winooski, VT, USA). Results were indicated as equivalent micrograms of L-leucine per mg of protein in the supernatants. 

### 2.6. Electrophoresis of Muscle Proteins

Protein fractions contained in SPP and MFP extracts were separated by SDS-PAGE according to [29] using a Mini Protean II electrophoresis chamber (Bio-Rad, Richmond, CA, USA), with 4% polyacrylamide stacking gels. For SPP and MFP, 15% and 12% separating gels were used respectively.

The extracts were diluted (1:1) in sample buffer (0.125 M Tris-HCl, 20% *v*/*v* glycerol, 0.04% *w*/*v* bromophenol blue, 2% *w*/*v* SDS, and 20% *v*/*v* β-mercaptoethanol), and boiled for 2 min. The mixtures were placed in each well so as to load 20 and 25 µg soluble protein of SPP and MFP extracts, respectively. In each gel were added five µL of standard molecular mass markers (Sigma-Aldrich, Madrid, Spain) containing myosin rabbit muscle (200.0 kDa), β-galactosidase *E. coli* (116 kDa), rabbit phosphorylase b (97 kDa), bovine albumin (66 kDa), ovalbumin from egg white (45.0 kDa), and bovine carbonic anhydrase (29.0 kDa).

Coomassie Brilliant Blue (BBC R-250) 0.1% (*w*/*v*) in methanol-acetic acid-water solution (40:10:50) was used to stain the gels overnight, and were then distained in the same solution without the stain. 

### 2.7. Densitometric Analysis

Densitometric analysis of gels was done with specific software (Image Analyser, Genesnap version 6.08, Synoptics) for assigning relative molecular masses to the SPP and MFP separated bands to quantify the relative contribution of each fraction to the total optical density of each lane, expressed as a percentage.

### 2.8. Statistical Analysis

For each parameter studied was determined the influence of “farm origin”, “transport duration”, and “ageing time”, and their interactions, by means of GLM analysis, using specific software (SPSS statistic, version 22.0). Least square means were tested for differences following Fisher’s least significant difference (LSD) procedure, and the strength of the effect of both categorical variables and their interaction on each of the measured parameters was estimated by means of the Fisher’s “F ratio” test. A significance level of 95% was considered to indicate statistical difference (*p* < 0.05). Results expressed as a percentage (e.g., densitometry) were normalized using the arcsine transformation of their square root.

## 3. Results

**Figure 1:**
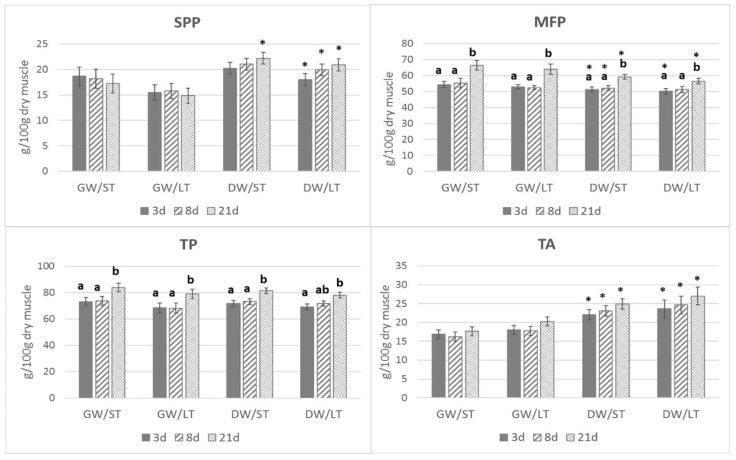
Effects of farm origin, transport time, and ageing time on sarcoplasmic (SPP), myofibrillar (MFP), total protein (TP) and total amino acids (TA) (g 100 g^−1^ dry muscle) contents of goat kids’ *Longissimus thoracis* muscle. Asterisks indicate significant differences attributable to farm origin (*p* < 0.05). Different letters show significant differences attributable to post-mortem time (*p* < 0.05). Values are average ± se. GW: high welfare-friendly management farm; DW: low welfare friendly management farm; ST: short transport; LT: long transport; 3d: 3 days post-mortem (dpm); 8d: 8 dpm; 21 d: 21 dpm. Significance of the three effects: SPP: *p* < 0.001 for farm origin; rest not significant. MFP: *p* < 0.01 for farm origin; *p* < 0.001 for ageing time; rest not significant. TP: *p* < 0.05 for transport duration; *p* < 0.01 for ageing time; rest not significant. TA: *p* < 0.001 for farm origin; rest not significant. Interactions: were not significant.

Values measured for the parameters of sarcoplasmic proteins, myofibrillar proteins, total proteins, and total amino acids are shown in Figure 1. The significance of the variables of farm origin, transport, and ageing time, as well as their interactions on the different parameters measured in muscle samples are summarized in the Figure 1 legend. Considering the values overall, the amount of muscle sarcoplasmic protein extracted tended to be higher in low, welfare-friendly management farm animals, although, considering each sampling time individually, significant differences were found only at 21 dpm in animals transported for 2 h (short transport duration), and at 3, 8, and 21 days in animals transported for 6 h (long transport duration). With regard to myofibrillar protein, overall concentration increased through ageing (*p* < 0.001), although low welfare-friendly management farm animals yielded significantly lower values for myofibrillar protein compared to good welfare-friendly management farms (*p* = 0.002). Total protein (TP) also increased during ageing and decreased in LT animals. Parameter total amino acids showed higher values in low welfare-friendly management farm animals (*p* < 0.001) compared to good welfare-friendly management farms. 

**Figure 2:**
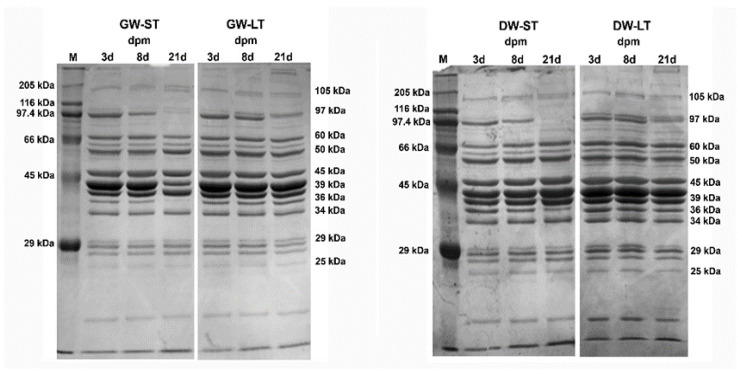
Effect of farm origin, transport duration, and ageing time on SDS-PAGE separation of sarcoplasmic protein (SPP) extracts. GW: high welfare-friendly management farm; DW: low welfare friendly management farm; ST: short transport; LT: long transport; 3d: 3 days post-mortem (dpm); 8d: 8 dpm; 21d: 21 dpm. M, molecular mass marker.

**Figure 3:**
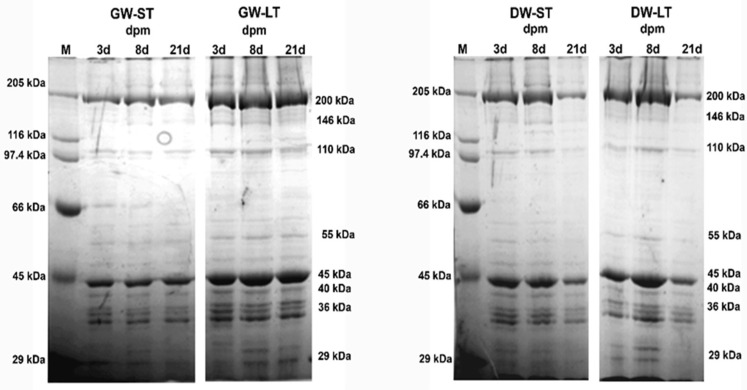
Effects of farm origin, transport duration and ageing time on SDS-PAGE separation of myofibrillar (MFP) extracts. GW: high welfare-friendly management farm; DW: low welfare-friendly management farm; ST: short transport; LT: long transport. 3d: 3 days post-mortem (dpm); 8d: 8 dpm; 21d: 21 dpm. M, molecular mass marker.

Figure 2 and Figure 3 showed sarcoplasmic and myofibrillar protein electrophoretic profiles from *Longissimus lumborum* muscle respectively. Quantification of the relative optical density (ROD) of each fraction, expressed as a % of total optical density, is shown in Table 1 and Table 2. Twelve major fractions were obtained from sarcoplasmic protein extracts (Figure 2) that were identified according to their molecular mass (Table 1) as B-97, B-65, B-60, B-58, B-46, B-43, B-40, B-38, B-36, B-30, B-28, B-26, B-24, and B-16 kDa. The fractions that contributed most to the optical density were those designed as B-46, B-43, and B-40, accounting together for 45% of total optical density. 

Considered together, significant differences in relative optical density were found owing to the factor farm origin (GW vs. DW) in B-97 (*p* = 0.008), B-46 (*p* = 0.004), B-38 (*p* = 0.001), B-36 (*p* = 0.016), and B-26 (*p* = 0.006). On the other hand, transport duration caused differences only in B-65 (*p* = 0.013), whereas ageing time had a significant effect on B-97 (*p* < 0.001), B-46 (*p* = 0.012), B-38 (*p* < 0.001), and B-26 (*p* < 0.001). In contrast, other sarcoplasmic protein fractions, namely B-43, B-30, B-28, and B-16 were not affected by any of the factors studied. 

For myofibrillar proteins, SDS-PAGE (Figure 3, Table 2) separated fractions were grouped (agreeing to values observed in previous literature) in this way: myosin heavy chain (MHC, 200 kDa), protein C (146 kDa), α-actinin (110 kDa), desmin (55 kDa), actin (45 kDa), troponin-T (Tn-T, 40 kDa), a group of fragments from 35 and 37 kDa, and another group of bands of low molecular mass (LMW, 26–30 kDa). MHC and actin were the most contributing fractions to total relative optical density, with approximately 30% of the total optical density each. 

Considered as a whole, farm origin (good welfare friendly management vs. low welfare-friendly management farm) was responsible for significant differences in relative optical density values of desmin (*p* = 0.016), Tn-T (*p* = 0.001), 35–37 kDa group (*p* = 0.001), and 29–30 kDa group (*p* < 0.001). Transport duration (short transport duration vs. long transport duration) only influenced protein C (*p* = 0.002), and the 35–37 kDa group (*p* = 0.020), whereas ageing time caused a significant effect on Tn-T (*p* = 0.001), the 35-37 kDa group (*p* = 0.001), and the 26–30 kDa group (*p* < 0.001). A significant decrease in the relative optical density of protein C, desmin, and TnT was observed in muscle extracts of animals from the deficient welfare farm. Conversely, the relative optical density of fractions 29–30 kDa and 35–37 kDa was higher in low welfare-friendly management farm animals, compared to good welfare-friendly management farms. The influence of ageing time was significant for TnT (40 kDa), which clearly decreased, and for fractions 26–30 kDa and 35–37 kDa, which increased irrespective of farm origin or transport time.

## 4. Discussion

### 4.1. Proteins and Aminoacids Content

Regarding farm origin, the results suggest that managing conditions of kids in low welfare-friendly management farms increased the post-mortem susceptibility of muscle proteins to degradation, according to the higher values observed for sarcoplasmic proteins (SPP), and total amino acids (TA), together with lower myofibrillar protein (MFP) content. The increase observed in sarcoplasmic protein fraction is likely attributable to the solubilization of structural proteins, which cannot be extracted in low ionic strength buffers when their structure is intact, but they could have contributed to increased sarcoplasmic protein fraction as a result of greater proteolysis. Simultaneous increase in total amino acids might confirm this hypothesis. However, this higher proteolysis observed in low welfare-friendly management farm animals was not reflected clearly in terms of meat quality characteristics, such as water holding capacity (WHC), or color as observed in previous studies, see [23]. Nevertheless, certain influence on *Longissimus lumborum* muscle hardness was observed as a consequence of a synergic effect of farm origin and transport time, pointing to higher toughness on muscle from low welfare-friendly management farm animals subjected to long transport duration treatment. Previous studies have shown reduced post-mortem proteolytic activity and imperceptible meat softening in goats.

With regard to the influence of transport time on these parameters, the results indicate that longer transport time before slaughtering caused no effect on sarcoplasmic proteins, myofibrillar proteins or total amino acid measurements. Considering both factors together, the results observed during meat ageing suggest that proteolysis of muscle proteins are less influenced by short-term stress than to poor handling conditions kept on the farm for longer.

The relative increase observed in myofibrillar protein and total protein contents during the post-mortem storage might well be attributed to an indirect consequence of carcass dehydration, such as described previously [30].

### 4.2. Electrophoresis of Sarcoplasmic Proteins

Some of the bands obtained coincided with those identified by [31] in pork (97 kDa fraction was identified as glycogen phosphorylase (GP); 58 kDa band as pyruvate kinase (PK); 46 kDa band as α-enolase (ENO1); 43 kDa as creatine kinase (CK); 40 kDa as aldolase; 38 kDa as lactate dehydrogenase (LDH); 36 kDa as glyceraldehyde 3-phosphate dehydrogenase (GAPDH); 26.5 kDa as triosephosphate isomerase (TPI); and 16.9 kDa as myoglobin).

The three variables considered in the study (farm origin, transport time, and post-mortem storage time) were responsible for changes in the electrophoretic pattern of sarcoplasmic proteins. Some studies have reported changes in sarcoplasmic proteins under stressful conditions in different species, a fact that enabled us to propose these proteins (most of them enzymes with a relevant role in muscle cell metabolism) as indicators of pre-slaughtering stress [6,14,15]. Thus, the last study found that *Longissimus thoracis* muscle in pigs under stressful pre-slaughtering transport showed a clear post-mortem decrease of some sarcoplasmic proteins, such as creatine kinase (CK), adenylate cyclase (AK), lactate dehydrogenase (LDH), and glyceraldehyde-3-phosphate dehydrogenase (GAPDH), indicating a depletion of these enzymes due to the effect of stress, caused by early depletion of muscle cells during post-mortem time. Similarly, [32] reported decreased solubility and activity of glycogen phosphorylase and creatine kinase enzymes in PSE pork meat. In contrast, it has been described that other protein fractions increase as a result of stressful management, such as the glycolytic enzyme ENO1 (46 kDa), both in bovines [33,34,35].

Our results indicate that the optical density of some fractions tended to be higher in animals raised in low welfare-friendly management farms, such as B-38, B-46, and B-43, whereas B-40 fraction increased with ageing time in high welfare-friendly management animals. Although some of the changes observed for sarcoplasmic protein proteins can be linked to the factor “farm origin”, from a practical point of view, and given that the relationship was not consistent under our experimental conditions, it is doubtful that a clear conclusion can be drawn.

With regard to the influence of ageing time on the integrity of muscle proteins, it is a well-known phenomenon that storage time is responsible for the degradation of both structural and metabolic proteins [5], a fact that enables us to establish these changes as relevant markers of tenderness in beef and pork. Thus, changes in 97 kDa, 43 kDa, and 38 kDa fragments are among the most relevant metabolic protein fractions linked to meat ageing in pork [5,9,10], having established that these enzymes are in turn preferential substrates of calpain, one of the main autolytic enzymes of sarcoplasm involved in meat maturation. Our study on goat meat confirmed that the ageing process affected 97 kDa and 38 kDa fractions, but no changes were detected for 43 kDa. With regard to the possible influence of on-farm managing conditions on post-mortem muscle ageing throughout the 21-d trial, both B97 and B38 fractions displayed significant differences as a result of the interaction of both variables. Hence, a sort of synergetic interaction seemed to have occurred for the B97 fraction whereby its relative optical density decreased in those animals from low welfare-friendly management farms transported for a longer period (long transport duration). Farm origin had a certain impact on B38 fraction during ageing, although in this specific case, relative optical density increased in low welfare-friendly management farm animals.

### 4.3. Electrophoresis of Myofibrillar Proteins

In the case of myofibrillar proteins, the structural proteins myosin heavy chain (MHC) and actin are acknowledged for their stability, and therefore, scarce changes are expected to occur during the storage in different species, such as bovines. Hence, these myofibrillar protein fractions are not regarded as valid indicators of meat softening. In agreement, no significant changes have been observed during post-mortem storage in our study for these two bands. Neither the variables farm origin nor transport duration yielded any effect on their relative optical density.

In contrast, clear modifications in relative optical density were observed in our study for other myofibrillar protein fractions, like troponin-T and desmin. These proteins have been described as sensitive protein fragments to post-mortem muscle degradation. Thus, troponin-T optical density was lower in low welfare-friendly management farm animals, no matter the transport duration or ageing time considered. This fraction tended to decrease throughout ageing time, in line with previous reports [36,37]. Conversely, the desmin (55 kDa) band was stable during ageing in our study, possibly due to the low proteolysis rate observed previously in the muscle of this species [38], although farm origin had an impact on this fraction, consisting in decreased relative optical density in animals from low welfare-friendly management farms compared to high welfare friendly management farm. This fact suggests a certain fragility of this fraction when on-farm management conditions are not adequate.

Post-mortem deterioration of desmin and troponin-T (38 kDa) have been described previously in pork meat [39]. Roughly speaking, such degradation was consistent with hardness measurements throughout meat aging, this suggesting the key role of both protein fractions in tenderness.

With regard to goat meat, disparate results have been reported on this specific subject, and thus, some studies observed that the optical density of troponin-T decreased during cold storage [36], in line with our results, whereas [38] showed no significant effects of aging time on troponin-T goat muscle. In the last study, however, desmin concentration tended to decrease after 4 days post-mortem, which partially agrees with our results. An increase in a 30-kDa peptide has been described for most livestock species in myofibrillar protein extracts during post-mortem storage, which is considered a degradation product of some myofibrillar components, such as troponin-T [35,40]. Therefore, this peptide is considered a marker of meat softening.

Our results indicate that a significant decrease in relative optical density for troponin took place, simultaneously to an increase in 26–30 kDa and 35–37 kDa bands, influenced by both farm origin and ageing time (Table 2). These results seem to confirm the validity of these fractions as indices of meat tenderness in goat kids meat, but also suggest their relationship with on-farm managing conditions (since the relative optical density measured for these fractions was significantly different between GW and low welfare-friendly management farm animals during the complete ageing period). No literature reporting changes in myofibrillar protein fractions attributable to deficient management on-farm has been found for this species. 

## 5. Conclusions

Electrophoretic separation of muscle sarcoplasmic and myofibrillar proteins provide valuable information relative to the influence of different factors, such as on-farm management, or transport duration, on the post-mortem processes taking place in the muscle of those animals. Both protein extracts displayed significant changes in some of their fractions (e.g., 97 kDa, 38 kDa, and 26 kDa for sarcoplasmic protein, and Troponin-T, 26–30 kDa and 35–37 kDa bands for myofibrillar protein) attributable to meat maturation, in line with the results obtained in similar or related studies carried out on different livestock species. The impact of on-farm management was consistently lower than that of “storage time”. In fact, it was not possible to identify any sarcoplasmic protein fraction whose variation could be clearly associated with on-farm management. On the contrary, with regard to myofibrillar proteins, the results indicate that troponin-T and other protein fractions (26–30 kDa and 35–37 kDa) were more influenced by farm of origin than by storage time. These results suggest that these protein fractions could be used as indicators of poor long-term management during the rearing of these animals. 

## Figures and Tables

**Table 1:** Effect of farm origin, transport duration time and ageing time on sarcoplasmic protein fractions extracted from *Longissimus lumborum*. Values (means ± SE; *n* = 4) indicate the relative optical density of each protein fraction (expressed as %) after SDS-PAGE separation and the significance of the three effects and their interaction.

		ST			LT									
	Farm Origin	Post-Mortem Time (days)			Post-Mortem Time (days)			FO	TD	AT	FO*TD	FO*AT	TD*AT	FO*TD*AT
		3	8	21	3	8	21							
**B-97 kDa**	GW	5.07 ^b^ ± 0.57	4.59 ^b^ ± 0.51	2.66 ^a^ ± 0.33	7.84 ^c#^ ± 0.45	5.35 ^b^ ± 0.46	1.89 ^a^ ± 0.51	*	ns	***	ns	*	ns	*
	DW	6.05 ^b^ ± 0.57	4.13 ^ab^ ± 0.51	3.03 ^a^ ± 0.33	5.21 ^b^* ± 0.45	3.72 ^ab^* ± 0.46	3.16 ^a^ ± 0.51							
**B-65 kDa**	GW	5.77 ± 0.53	5.52 ± 0.58	5.99 ± 0.47	7.02 ± 0.40	7.07 ± 0.38	6.38 ± 0.40	ns	*	ns	ns	ns	ns	ns
	DW	5.44 ± 0.53	6.13 ± 0.58	7.14 ± 0.47	6.59 ± 0.40	6.36 ± 0.38	6.77 ± 0.40							
**B-58 kDa**	GW	8.34 ± 0.17	7.45 ± 0.68	8.48 ± 0.19	7.66 ^a^ ± 0.69	9.24 ^b^ ± 0.58	9.88 ^b#^ ± 0.48	ns	ns	ns	*	ns	ns	ns
	DW	8.24 ± 0.19	9.04 ± 0.68	8.44 ± 0.22	8.47 ± 0.69	7.81 ± 0.58	8.51 ± 0.48							
**B-46 kDa**	GW	10.25 ± 0.49	9.07 ± 0.50	9.71 ± 0.46	9.75 ± 0.49	10.09 ± 0.73	11.85 ^#^ ± 0.62	**	ns	*	*	ns	ns	ns
	DW	11.00 ± 0.49	11.12 * ± 0.50	12.49 * ± 0.46	10.17 ± 0.49	10.54 ± 0.73	11.40 ± 0.62							
**B-43 kDa**	GW	23.45 ± 0.81	21.43 ± 1.08	20.80 ± 0.32	21.37 ± 1.54	22.17 ± 1.48	19.06 ± 0.58	ns	ns	ns	ns	ns	ns	ns
	DW	25.84 ± 0.93	22.95 ± 1.08	21.59 ± 0.28	21.91 ± 1.54	21.09 ± 1.48	22.56 ^#^* ± 0.58							
**B-40 kDa**	GW	11.88 ± 1.13	11.61 ± 0.91	13.93 ± 1.64	12.14 ^a^ ± 1.02	11.60 ^a^ ± 0.85	15.74 ^b^ ± 0.65	ns	ns	ns	ns	ns	ns	ns
	DW	12.07 ± 1.13	10.81 ± 0.91	10.74 ± 1.64	11.60 ± 1.02	11.66 ± 0.85	12.17 * ± 0.65							
**B-38 kDa**	GW	6.59 ^c^ ± 0.66	4.47 ^b^ ± 0.27	1.74 ^a^ ± 0.24	6.51 ^b^ ± 0.46	5.45 ^b^ ± 0.54	1.52 ^a^ ± 0.40	**	ns	***	ns	**	ns	ns
	DW	7.37 ^c^ ± 0.66	5.88 ^b^* ± 0.24	3.38 ^a^* ± 0.24	6.14 ^c^ ± 0.40	5.20 ^ab^ ± 0.54	3.98 ^a^* ± 0.40							
**B-36 kDa**	GW	7.47 ± 0.59	7.50 ± 0.70	7.48 ± 0.52	5.73 ^a^ ± 0.47	6.48 ^a^ ± 0.49	8.54 ^b^ ± 0.50	*	ns	ns	ns	ns	ns	ns
	DW	6.64 ± 0.59	6.84 ± 0.70	6.48 ± 0.52	6.13 ± 0.40	6.16 ± 0.49	6.78 ± 0.50							
**B-30 kDa**	GW	1.96 ± 0.67	2.17 ± 0.37	2.41 ± 0.26	2.33 ± 0.17	2.75 ± 0.38	2.16 ± 0.38	ns	ns	ns	ns	ns	ns	ns
	DW	2.10 ± 0.67	2.05 ± 0.37	2.24 ± 0.26	2.39 ± 0.15	2.94 ± 0.38	2.33 ± 0.38							
**B-28 kDa**	GW	4.33 ± 0.38	5.41 ± 0.25	4.45 ± 0.18	4.14 ± 0.26	4.60 ± 0.32	5.02 ± 0.50	ns	ns	ns	ns	ns	ns	ns
	DW	4.75 ± 0.38	4.70 ± 0.21	4.75 ± 0.15	5.01 ± 0.26	4.91 ± 0.28	5.36 ± 0.50							
**B-26 kDa**	GW	2.25 ^a^ ± 0.29	4.33 ^ab^ ± 0.30	4.56 ^b^ ± 0.46	2.58 ^a^ ± 0.31	3.39 ^b^ ± 0.39	4.80 ^b^ ± 0.47	**	ns	***	ns	ns	ns	ns
	DW	2.05 ^a^ ± 0.29	2.71 ^a^* ± 0.26	3.98 ^b^ ± 0.40	2.23 ^a^ ± 0.27	3.58 ^b^ ± 0.34	3.74 ^b^ ± 0.47							
**B-16 kDa**	GW	2.67 ± 0.48	3.04 ± 0.24	2.10 ± 0.44	3.17 ± 0.32	3.54 ± 0.41	2.54 ± 0.39	ns	ns	ns	ns	ns	ns	ns
	DW	2.75 ± 0.48	2.30 ± 0.21	2.83 ± 0.44	3.11 ± 0.32	2.56 ± 0.41	2.47 ± 0.39							

Asterisks indicate significant differences attributable to farm origin (*p* < 0.05). # shows significant differences attributable to transport (*p* < 0.05). Lower case superscripts show significant differences attributable to post-mortem time (*p* < 0.05). Values are mean ± se. **GW**: high welfare-friendly management farm; **DW**: low welfare-friendly management farm; **ST**: short transport duration; **LT**: long transport duration. **FO**: farm origin; **TD**: transport duration; **AT**: muscle ageing time. n.s. = not significant; * *p* < 0.05. ** *p* < 0.01. *** *p* < 0.001.

**Table 2:** Effect of farm origin, transport duration time and ageing time on myofibrillar protein fractions extracted from *Longissimus lumborum.* Values (means ± SE; *n* = 4) indicate the relative optical density of each protein fraction (expressed as %) after SDS-PAGE separation and the significance of the three effects and their interaction.

		ST			LT									
	Farm Origin	Post-Mortem Time (days)			Post-Mortem Time (days)			FO	TD	AT	FO*TD	FO*AT	TD*AT	FO*TD*AT
		3	8	21	3	8	21							
**MHC †**	GW	31.05 ± 0.65	30.48 ± 0.53	31.42 ± 0.91	31.76 ± 0.23	31.25 ± 0.26	30.40 ± 0.56	ns	ns	ns	ns	ns	ns	ns
	DW	30.83 ± 0.65	29.87 ± 0.62	29.74 ± 0.91	31.05 ^b^ ± 0.26	29.99 ^a^ ± 0.22	29.82 ^a^ ± 0.56							
**Protein C ‡**	GW	1.42 ± 0.32	1.11 ± 0.26	1.11 ± 0.23	1.91 ± 0.20	2.05 ± 0.41	1.93 ± 0.12	ns	**	ns	ns	ns	ns	ns
	DW	1.66 ± 0.32	0.85 ± 0.30	0.97 ± 0.23	1.64 * ± 0.20	1.12 * ± 0.41	1.19 * ± 0.12							
**α-actinin §**	GW	2.89 ± 0.27	2.55 ± 0.35	2.38 ± 0.21	2.65 ± 0.20	2.32 ± 0.16	2.41 ± 0.18	ns	ns	ns	ns	ns	ns	ns
	DW	1.88 ± 0.27	2.04 ± 0.35	2.67 ± 0.21	2.51 ± 0.20	2.74 ± 0.29	2.07 ± 0.18							
**Desmin**	GW	2.75 ± 0.17	1.98 ± 0.13	2.21 ± 0.28	2.39 ± 0.13	1.53 ± 0.32	2.07 ± 0.24	*	ns	ns	ns	ns	ns	ns
	DW	1.99 * ± 0.17	1.63 * ± 0.11	1.45 * ± 0.28	1.71 * ± 0.23	1.58 ± 0.32	1.55 ± 0.24							
**Actin ϒ**	GW	31.03 ± 0.23	31.08 ± 0.60	30.28 ± 0.32	31.77 ± 0.34	30.97 ± 0.53	31.16 ± 0.30	ns	ns	ns	ns	ns	ns	ns
	DW	30.59 ± 0.27	30.50 ± 0.60	29.40 ± 0.32	30.91 ± 0.34	29.88 ± 0.53	29.07 ± 0.26							
**TnT Ϯ**	GW	8.47 ^b^ ± 0.69	5.94 ^a^ ± 0.69	6.04 ^a^ ± 0.69	7.94 ^b^ ± 0.36	6.68 ^a^ ± 0.36	6.10 ^a^ ± 0.36	***	ns	***	ns	ns	ns	ns
	DW	5.85 ^b^* ± 0.45	3.74 ^a^* ± 0.45	4.37 ^ab^* ± 0.45	5.32 * ± 0.65	4.34 * ± 0.65	4.57 * ± 0.65							
**35–37 kDa group ψ**	GW	9.96 ^a^ ± 0.57	12.88 ^b^ ± 0.57	13.40 ^b^ ± 0.57	9.85 ^a^ ± 0.89	12.31 ^b^ ± 0.89	10.84 ^b^ ± 0.89	***	*	**	ns	ns	ns	ns
	DW	14.76 * ± 0.46	16.30 * ± 0.46	15.58 * ± 0.46	12.68 ^a#^* ± 0.83	14.86 ^b*^ ± 0.83	15.10 ^b^* ± 0.83							
**26–30 kDa** **Group ϐ**	GW	11.91 ^a^ ± 0.48	13.20 ^ab^ ± 0.51	14.21 ^b^ ± 0.42	11.8 ^a^ ± 0.52	13.9 ^b^ ± 0.58	14.2 ^b^ ± 0.46	***	ns	***	ns	ns	ns	ns
	DW	13.10 ^a^ ± 0.48	16.12 ^b^* ± 0.51	15.90 ^b^* ± 0.42	12.3 ^a^ ± 0.52	15.7 ^b^* ± 0.58	16.6 ^b^* ± 0.46							

Asterisks indicate significant differences attributable to farm origin (*p* < 0.05). # shows significant differences attributable to transport (*p* < 0.05). Lower case superscripts show significant differences attributable to post-mortem time (*p* < 0.05). Values are mean ± se. **GW**: high welfare-friendly management farm; **DW**: low welfare-friendly management farm; **ST**: short transport duration; **LT**: long transport duration. **FO**: farm origin; **TD**: transport duration; **AT**: muscle ageing time. n.s. = not significant; * *p* < 0.05. ** *p* < 0.01. *** *p* < 0.001. † MHC: 205 kDa protein fraction designed as myosin heavy chain. ‡ 146 kDa protein fraction designed as Protein C. § 110 kDa protein fraction designed as α-actinin. 55 kDa protein fraction designed as desmin. ϒ 45 kDa protein fraction designed as actin. Ϯ 38 kDa protein fraction designed as Troponin T. ψ Group bands ranging from 35–37 kDa. ϐ protein fraction 26–30 kDa.
